# (2*Z*,2′*Z*)-Diethyl 3,3′-[butane-1,4-diylbis(aza­nedi­yl)]bis­(but-2-enoate)

**DOI:** 10.1107/S1600536812036823

**Published:** 2012-09-05

**Authors:** Mohamed Anouar Harrad, Brahim Boualy, Mustapha Ait Ali, Larbi El Firdoussi, Helen Stoeckli-Evans

**Affiliations:** a Equipe de Chimie de Coordination, Faculté des Sciences Semlalia, BP 2390, Marrakech, Morocco; b Institute of Physics, University of Neuchâtel, 2000 Neuchâtel, Switzerland

## Abstract

The whole mol­ecule of the title β-enamino­ester, C_16_H_28_N_2_O_4_, is generated by a crystallographic inversion center, situated at the mid-point of the central C—C bond of the 1,4-diamino­butane segment. There are two intra­molecular N—H⋯O hydrogen bonds that generate *S*(6) ring motifs. This leads to the *Z* conformation about the C=C bonds [1.3756 (17) Å]. The mol­ecule is S-shaped with the planar central 1,4-diamino­butane segment [maximum deviation for non H-atoms = 0.0058 (13) Å] being inclined to the ethyl butyl­enonate fragment [C—C—O—C—C=C—C; maximum deviation = 0.0710 (12) Å] by 15.56 (10)°. In the crystal, mol­ecules are linked *via* C—H⋯O inter­actions, leading to the formation of an undulating two-dimensional network lying parallel to the *bc* plane.

## Related literature

For general background to the use of β-enamino esters as precursors in organic synthesis, see: Eddington *et al.* (2000 ▶); Palmieri & Cimarelli (1996 ▶); Zhang & Hu (2006 ▶). For the synthesis of β-enamino esters, see: Harrad *et al.* (2010 ▶); Hegde & Jones (1993 ▶); Lue & Greenhill (1997 ▶); Katritzky *et al.* (2004 ▶); Bartoli *et al.* (1995 ▶); Reddy *et al.* (2005 ▶). For the structure of related compounds, see: Harrad *et al.* (2011*a* ▶,*b* ▶); Amézquita-Valencia *et al.* (2009 ▶). For hydrogen-bond motifs, see: Bernstein *et al.* (1995 ▶).
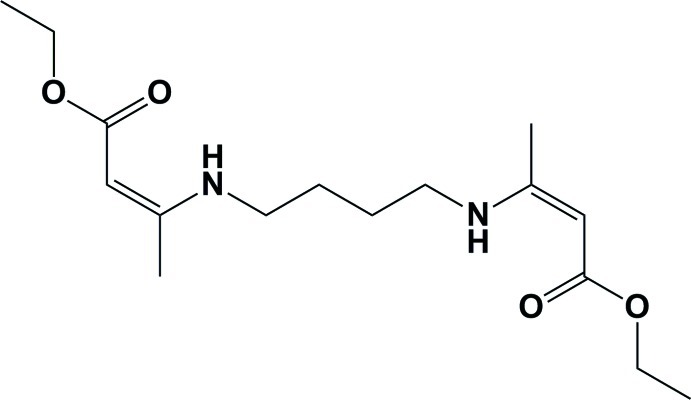


## Experimental

### 

#### Crystal data

C_16_H_28_N_2_O_4_
*M* *_r_* = 312.40
Monoclinic, 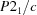

*a* = 5.7624 (5) Å
*b* = 13.1329 (8) Å
*c* = 11.7601 (9) Å
β = 98.547 (6)°
*V* = 880.09 (12) Å^3^
*Z* = 2
Mo *K*α radiation
μ = 0.08 mm^−1^
*T* = 133 K
0.45 × 0.40 × 0.30 mm

#### Data collection

Stoe IPDS 2 diffractometer
Absorption correction: multi-scan (MULscanABS in *PLATON*; Spek, 2009 ▶) *T*_min_ = 0.679, *T*_max_ = 1.000
9379 measured reflections
1661 independent reflections
1392 reflections with *I* > 2σ(*I*)
*R*_int_ = 0.052

#### Refinement

*R*[*F* ^2^ > 2σ(*F* ^2^)] = 0.035
*wR*(*F* ^2^) = 0.081
*S* = 1.04
1661 reflections
107 parameters
H atoms treated by a mixture of independent and constrained refinement
Δρ_max_ = 0.15 e Å^−3^
Δρ_min_ = −0.14 e Å^−3^

### 

Data collection: *X-AREA* (Stoe & Cie, 2009 ▶); cell refinement: *X-AREA*; data reduction: *X-RED32* (Stoe & Cie, 2009 ▶); program(s) used to solve structure: *SHELXS97* (Sheldrick, 2008 ▶); program(s) used to refine structure: *SHELXL97* (Sheldrick, 2008 ▶); molecular graphics: *PLATON* (Spek, 2009 ▶) and *Mercury* (Macrae *et al.*, 2008 ▶); software used to prepare material for publication: *SHELXL97*, *PLATON* and *publCIF* (Westrip, 2010 ▶).

## Supplementary Material

Crystal structure: contains datablock(s) I, global. DOI: 10.1107/S1600536812036823/ds2210sup1.cif

Structure factors: contains datablock(s) I. DOI: 10.1107/S1600536812036823/ds2210Isup2.hkl

Supplementary material file. DOI: 10.1107/S1600536812036823/ds2210Isup3.cml

Additional supplementary materials:  crystallographic information; 3D view; checkCIF report

## Figures and Tables

**Table 1:** Hydrogen-bond geometry (Å, °)

*D*—H⋯*A*	*D*—H	H⋯*A*	*D*⋯*A*	*D*—H⋯*A*
N1—H1*N*⋯O2	0.880 (16)	2.000 (16)	2.7099 (14)	136.9 (13)
C8—H8*C*⋯O2^i^	0.98	2.51	3.4697 (16)	167

Symmetry code: (i) 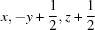.

**Fig. 1.**
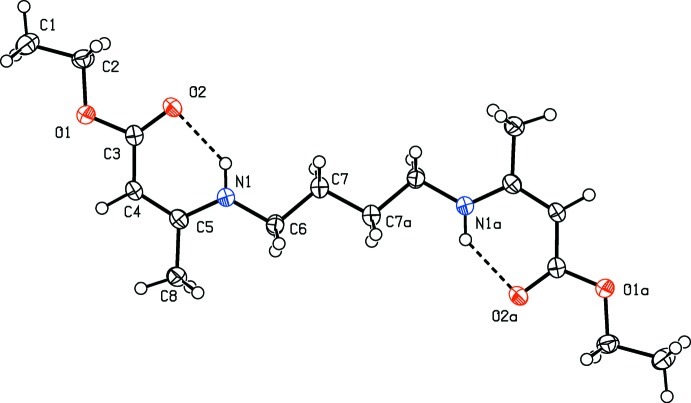
The molecular structure of the title molecule, showing the atom numbering. The displacement ellipsoids are drawn at the 50% probability level [symmetry code: (a) = -x, -y, -z].

**Fig. 2.**
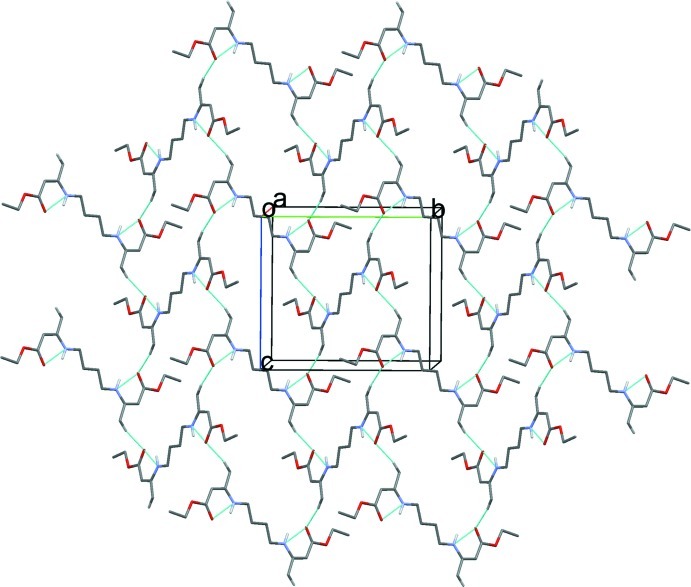
A view along the a axis of the crystal packing of the title compound, showing the N—H···O and C—H···O hydrogen bonds as dashed cyan lines.

## supplementary crystallographic information

### Comment

β-Enamino esters are useful precursors for the preparation of biologically active compounds such as β-amino acids and γ-amino alcohols (Eddington *et al.*, 2000; Palmieri & Cimarelli, 1996; Zhang & Hu, 2006). Many synthetic methods have been developed for the preparation of these compounds (Harrad *et al.*, 2010, 2011*a*,*b*; Hegde & Jones, 1993; Lue & Greenhill, 1997; Katritzky *et al.*, 2004; Bartoli *et al.*, 1995; Reddy *et al.*, 2005). As part of our ongoing program focused on developing new methodologies for the synthesis of β-enamino compounds, we report herein on the synthesis and crystal structure of the title compound, a new β-di-enamino-di-ester.

The tile compound was prepared by condensation of ethyl 3-oxobutanoate with 1,4-diaminobutane using a catalytic amount of Ca(CF_3_COO)_2_ under solvent-free conditions according to the procedure we have previously described (Harrad *et al.*, 2010). The β-enaminoester was typically characterized by ^1^H, ^13^C NMR, IR and mass spectroscopy. The characteristic broad singlet for the amine proton appears at 8.50 p.p.m., the singlet corresponding to the proton of the double bond at 4.29 p.p.m. The triplet and the quartet for the ethyl moiety appeared at 1.11 and 3.98 p.p.m., respectively.

The molecular structure of the title molecule is illustrated in Fig. 1. It possesses a crystallographic inversion center situated at the middle of the central C7—C7a bond of the 1,4-diaminobutane segment [symmetry code: (*a*) = -*x*, -*y*, -*z*]. The bond distances and angles are normal and similar to those in related compounds (Harrad *et al.*, 2011*a*,*b*; Amézquita-Valencia *et al.*, 2009). There are two intramolecular N—H···O hydrogen bonds (Table 1), that generate S(6) ring motifs (Bernstein *et al.*, 1995). This leads to the *Z* conformation about the C4═C5 and C4a═C5a bonds (Fig. 1).

In the crystal, molecules are linked *via* C—H···O interactions leading to the formation of an undulating two-dimensional network that lies parallel to the *bc* plane (Table 1 and Fig. 2).

### Experimental

In a typical experiment 1.0 mmol of Ethyl acetoacetate, 0.5 mmol of 1,4-diaminobutane and 0.1 mmol of Ca(CF_3_COO)_2_ were stirred at room temperature for 30 min under solvent free conditions. At the end of the reaction, 10 ml of distilled water was added to the residue and it was extracted with diethyl ether (3 × 25 ml). The organic layer was dried over Na_2_SO_4_. The solvent was removed under reduced pressure, and pure β-enaminone was obtained by column chromatography over silica gel using hexane/ethyl acetate (5:95, *v*/*v*) as colourless block-like crystals on slow evaporation of the solvents [Yield 78%; *M*.p.: 435 - 437 K]. Spectroscopic data for the title compound: FT—IR (KBr, cm^-1^): 1655, 1607. ^1^H RMN (300 MHz, CDCl_3_) = 1.11 (t, J = 7.2 Hz, 6H, CH_3_—CH_2_—O), 1.56 (m, 4H, CH_2_—CH_2_—NH), 1.81 (s, 6H, CH_3_—C═CH), 3.16 (m, 4H, CH_2_—CH_2_—NH), 3.98 (q, J = 7.2 Hz, 4H, CH_3_—CH_2_—O), 4.29 (s, 2H, CH_3_—C═CH), 8.50 (br s, 1H, NH); ^13^C RMN (75 MHz, CDCl_3_) = 169.99 (–C═O), 160.78 (–C), 82.50 (–CH), 57.72 (O—CH_2_), 42.52 (CH_2_—NH), 27.84(CH_2_—C), 18.92 (CH_3_—C), 14.48 (CH_3_—C—O). MS (EI, 70 eV): m/*z* = 312 [*M*+].

### Refinement

All the H-atoms were located in a difference Fourier map. In the final cycles of refinement the NH H-atom was freely refined, while the C-bound H-atoms were included in calculated positions and treated as riding atoms: C—H = 0.95, 0.99 and 0.98 Å for CH(allyl), CH_2_ and CH_3_, respectively, with *U*_iso_(H) = k × *U*_eq_(C), where k = 1.5 for CH_3_ H-atoms, and = 1.2 for all other H-atoms.

### Crystal data

**Table:**

C_16_H_28_N_2_O_4_	*F*(000) = 340
*M**_r_* = 312.40	*D*_x_ = 1.179 Mg m^−^^3^
Monoclinic, *P*2_1_/*c*	Mo *K*α radiation, λ = 0.71073 Å
Hall symbol: -P 2ybc	Cell parameters from 8450 reflections
*a* = 5.7624 (5) Å	θ = 2.3–26.2°
*b* = 13.1329 (8) Å	µ = 0.08 mm^−^^1^
*c* = 11.7601 (9) Å	*T* = 133 K
β = 98.547 (6)°	Block, colourless
*V* = 880.09 (12) Å^3^	0.45 × 0.40 × 0.30 mm
*Z* = 2	

### Data collection

**Table:**

Stoe IPDS 2 diffractometer	1661 independent reflections
Radiation source: fine-focus sealed tube	1392 reflections with *I* > 2σ(*I*)
Plane graphite monochromator	*R*_int_ = 0.052
φ + ω scans	θ_max_ = 25.7°, θ_min_ = 2.3°
Absorption correction: multi-scan (MULscanABS in *PLATON*; Spek, 2009)	*h* = −7→7
*T*_min_ = 0.679, *T*_max_ = 1.000	*k* = −14→15
9379 measured reflections	*l* = −14→14

### Refinement

**Table:**

Refinement on *F*^2^	Secondary atom site location: difference Fourier map
Least-squares matrix: full	Hydrogen site location: inferred from neighbouring sites
*R*[*F*^2^ > 2σ(*F*^2^)] = 0.035	H atoms treated by a mixture of independent and constrained refinement
*wR*(*F*^2^) = 0.081	*w* = 1/[σ^2^(*F*_o_^2^) + (0.0346*P*)^2^ + 0.1872*P*] where *P* = (*F*_o_^2^ + 2*F*_c_^2^)/3
*S* = 1.04	(Δ/σ)_max_ < 0.001
1661 reflections	Δρ_max_ = 0.15 e Å^−^^3^
107 parameters	Δρ_min_ = −0.14 e Å^−^^3^
0 restraints	Extinction correction: *SHELXL97* (Sheldrick, 2008), Fc^*^=kFc[1+0.001xFc^2^λ^3^/sin(2θ)]^-1/4^
Primary atom site location: structure-invariant direct methods	Extinction coefficient: 0.015 (4)

### Special details

**Table:**

Geometry. Bond distances, angles *etc*. have been calculated using the rounded fractional coordinates. All su's are estimated from the variances of the (full) variance-covariance matrix. The cell e.s.d.'s are taken into account in the estimation of distances, angles and torsion angles
Refinement. The NH H-atom was located in a difference electron-density map and freely refined. The C-bound H-atoms were included in calculated positions and treated as riding atoms: C—H = 0.95, 0.99 and 0.98, Å for CH(allyl), CH_2_ and CH_3_ H-atoms, respectively, with *U*_iso_(H) = k × *U*_eq_(parent C-atom), where k = 1.5 for CH_3_ H-atoms and = 1.2 for other H atoms.

### Fractional atomic coordinates and isotropic or equivalent isotropic displacement parameters (Å^2^)

**Table:**

	*x*	*y*	*z*	*U*_iso_*/*U*_eq_	
O1	0.93675 (15)	0.33513 (7)	0.19771 (7)	0.0277 (3)	
O2	0.72096 (15)	0.23004 (7)	0.07166 (7)	0.0282 (3)	
N1	0.37954 (19)	0.12458 (8)	0.15569 (9)	0.0252 (3)	
C1	1.2379 (2)	0.44490 (11)	0.15191 (13)	0.0358 (4)	
C2	1.0631 (2)	0.36486 (10)	0.10551 (12)	0.0312 (4)	
C3	0.7649 (2)	0.26399 (9)	0.17015 (10)	0.0231 (3)	
C4	0.6507 (2)	0.23715 (9)	0.26565 (10)	0.0237 (3)	
C5	0.4629 (2)	0.17140 (9)	0.25516 (10)	0.0231 (3)	
C6	0.1806 (2)	0.05468 (10)	0.13799 (11)	0.0274 (4)	
C7	0.1049 (2)	0.03591 (10)	0.01049 (11)	0.0277 (4)	
C8	0.3384 (2)	0.15152 (11)	0.35620 (11)	0.0299 (4)	
H1A	1.15460	0.50510	0.17410	0.0540*	
H1B	1.33290	0.46370	0.09260	0.0540*	
H1C	1.34010	0.41820	0.21930	0.0540*	
H1N	0.458 (3)	0.1381 (11)	0.0988 (13)	0.033 (4)*	
H2A	0.95300	0.39230	0.04010	0.0370*	
H2B	1.14480	0.30530	0.07830	0.0370*	
H4	0.70620	0.26570	0.33890	0.0280*	
H6A	0.04810	0.08390	0.17190	0.0330*	
H6B	0.22510	−0.01070	0.17720	0.0330*	
H7A	0.23740	0.00610	−0.02300	0.0330*	
H7B	0.06300	0.10160	−0.02860	0.0330*	
H8A	0.32040	0.07790	0.36570	0.0450*	
H8B	0.18330	0.18370	0.34310	0.0450*	
H8C	0.43020	0.17990	0.42580	0.0450*	

### Atomic displacement parameters (Å^2^)

**Table:**

	*U*^11^	*U*^22^	*U*^33^	*U*^12^	*U*^13^	*U*^23^
O1	0.0308 (5)	0.0273 (5)	0.0257 (5)	−0.0067 (4)	0.0069 (4)	−0.0027 (4)
O2	0.0339 (5)	0.0306 (5)	0.0198 (4)	−0.0040 (4)	0.0030 (4)	−0.0016 (4)
N1	0.0257 (6)	0.0282 (6)	0.0215 (5)	−0.0047 (4)	0.0026 (4)	−0.0014 (4)
C1	0.0339 (7)	0.0320 (8)	0.0439 (8)	−0.0050 (6)	0.0133 (6)	−0.0005 (6)
C2	0.0361 (7)	0.0287 (7)	0.0305 (7)	−0.0037 (6)	0.0110 (6)	0.0022 (5)
C3	0.0240 (6)	0.0198 (6)	0.0242 (6)	0.0023 (5)	−0.0002 (5)	−0.0004 (5)
C4	0.0262 (6)	0.0251 (6)	0.0191 (6)	−0.0005 (5)	0.0008 (5)	−0.0028 (5)
C5	0.0242 (6)	0.0232 (6)	0.0211 (6)	0.0044 (5)	0.0006 (5)	0.0007 (5)
C6	0.0270 (6)	0.0273 (7)	0.0266 (7)	−0.0049 (5)	−0.0002 (5)	0.0000 (5)
C7	0.0280 (6)	0.0275 (7)	0.0269 (7)	−0.0034 (5)	0.0016 (5)	−0.0034 (5)
C8	0.0277 (7)	0.0375 (7)	0.0242 (7)	−0.0025 (6)	0.0033 (5)	−0.0004 (5)

### Geometric parameters (Å, º)

**Table:**

O1—C2	1.4467 (16)	C1—H1B	0.9800
O1—C3	1.3650 (15)	C1—H1C	0.9800
O2—C3	1.2318 (14)	C2—H2A	0.9900
N1—C5	1.3454 (16)	C2—H2B	0.9900
N1—C6	1.4592 (16)	C4—H4	0.9500
N1—H1N	0.880 (16)	C6—H6A	0.9900
C1—C2	1.5013 (19)	C6—H6B	0.9900
C3—C4	1.4277 (17)	C7—H7A	0.9900
C4—C5	1.3756 (17)	C7—H7B	0.9900
C5—C8	1.4994 (17)	C8—H8A	0.9800
C6—C7	1.5182 (18)	C8—H8B	0.9800
C7—C7^i^	1.5243 (17)	C8—H8C	0.9800
C1—H1A	0.9800		
			
C2—O1—C3	115.78 (9)	C1—C2—H2A	110.00
C5—N1—C6	125.66 (11)	C1—C2—H2B	110.00
C5—N1—H1N	114.3 (10)	H2A—C2—H2B	108.00
C6—N1—H1N	120.0 (10)	C3—C4—H4	119.00
O1—C2—C1	107.56 (11)	C5—C4—H4	119.00
O1—C3—C4	112.65 (10)	N1—C6—H6A	110.00
O1—C3—O2	120.77 (10)	N1—C6—H6B	110.00
O2—C3—C4	126.58 (11)	C7—C6—H6A	110.00
C3—C4—C5	122.20 (11)	C7—C6—H6B	110.00
N1—C5—C8	117.28 (11)	H6A—C6—H6B	108.00
N1—C5—C4	122.64 (11)	C6—C7—H7A	109.00
C4—C5—C8	120.06 (11)	C6—C7—H7B	109.00
N1—C6—C7	110.33 (10)	H7A—C7—H7B	108.00
C6—C7—C7^i^	111.40 (10)	C7^i^—C7—H7A	109.00
C2—C1—H1A	109.00	C7^i^—C7—H7B	109.00
C2—C1—H1B	109.00	C5—C8—H8A	109.00
C2—C1—H1C	109.00	C5—C8—H8B	109.00
H1A—C1—H1B	109.00	C5—C8—H8C	109.00
H1A—C1—H1C	109.00	H8A—C8—H8B	109.00
H1B—C1—H1C	109.00	H8A—C8—H8C	109.00
O1—C2—H2A	110.00	H8B—C8—H8C	110.00
O1—C2—H2B	110.00		
			
C3—O1—C2—C1	178.47 (10)	O1—C3—C4—C5	176.01 (11)
C2—O1—C3—O2	−1.34 (16)	O2—C3—C4—C5	−3.6 (2)
C2—O1—C3—C4	179.00 (10)	C3—C4—C5—N1	2.77 (19)
C6—N1—C5—C4	−179.53 (11)	C3—C4—C5—C8	−175.62 (11)
C6—N1—C5—C8	−1.09 (18)	N1—C6—C7—C7^i^	−179.28 (10)
C5—N1—C6—C7	167.36 (11)	C6—C7—C7^i^—C6^i^	179.97 (16)

Symmetry code: (i) −*x*, −*y*, −*z*.

### Hydrogen-bond geometry (Å, º)

**Table:**

*D*—H···*A*	*D*—H	H···*A*	*D*···*A*	*D*—H···*A*
N1—H1*N*···O2	0.880 (16)	2.000 (16)	2.7099 (14)	136.9 (13)
C8—H8*C*···O2^ii^	0.98	2.51	3.4697 (16)	167

Symmetry code: (ii) *x*, −*y*+1/2, *z*+1/2.
